# Reincentivizing – a new theory of work and work absence

**DOI:** 10.1186/1472-6963-7-100

**Published:** 2007-07-03

**Authors:** Hans O Thulesius, Birgitta E Grahn

**Affiliations:** 1 Department of Clinical Sciences Malmö, Division of Family Medicine, Lund University, Sweden; 2 Department of Health Sciences, Division of Physiotherapy, Lund University, Sweden; 3 R&D Welfare of Southern Smaland, Box 1223, SE-351 12 Växjö, Sweden

## Abstract

**Background:**

Work capacity correlates weakly to disease concepts, which in turn are insufficient to explain sick leave behavior. With data mainly from Sweden, a welfare state with high sickness absence rates, our aim was to develop an explanatory theory of how to understand and deal with work absence and sick leave.

**Methods:**

We used classic grounded theory for analyzing data from >130 interviews with people working or on sick leave, physicians, social security officers, and literature. Several hundreds of typed and handwritten memos were the basis for writing up the theory.

**Results:**

In this paper we present a theory of work incentives and how to deal with work absence. We suggest that work disability can be seen as hurt work drivers or people caught in mode traps. Work drivers are specified as work capacities + work incentives, monetary and non-monetary. Also, people can get trapped in certain modes of behavior through changed capacities or incentives, or by inertia. Different modes have different drivers and these can trap the individual from reincentivizing, ie from going back to work or go on working. Hurt drivers and mode traps are recognized by driver assessments done on several different levels. Mode driver calculations are done by the worker. Then follows employer, physician, and social insurance officer assessments. Also, driver assessments are done on the macro level by legislators and other stakeholders. Reincentivizing is done by different repair strategies for hurt work drivers such as body repair, self repair, work-place repair, rehumanizing, controlling sick leave insurance, and strengthening monetary work incentives. Combinations of these driver repair strategies also do release people from mode traps.

**Conclusion:**

Reincentivizing is about recognizing hurt work drivers and mode traps followed by repairing and releasing the same drivers and traps. Reincentivizing aims at explaining what is going on when work absence is dealt with and the theory may add to social psychological research on work and work absence, and possibly inform sick leave policies.

## Background

The sickness absence rate in Sweden is one of the highest in the world [1]. Sweden has generous sick leave policies and strong job protection legislation. There is no upper time limit for sick leave, and a low risk of loosing employment due to sickness absence. Monetary compensation from social security limits the loss of buying power to 0–20% after tax for people with low to average incomes on sick leave [2]. A too soft and disincentivizing social security system was a central political issue leading to a shift in Swedish government in 2006.

In welfare states such as Sweden non-monetary work incentives such as plight and pride seem more important than in laissez-faire economies such as the USA [2,3], where monetary work incentives are stronger [4]. Although Swedish sick leave compensation is generous a sick leave trajectory often involves shame and distrust. Against this background common disease concepts are inadequate to explain sick leave behavior since work capacity alone shows little correlation to disease severity [5-7]. Therefore our aim was to generate an explanatory theory of work absence and sick leave and how to deal with it.

## Methods

### Participants

Data collection started in 2003. We did 20 formal and 42 informal interviews with people working and on sick leave, informal interviews with 30 Swedish health care professionals (nurses, physicians and physiotherapists), and formal interviews and focus group interviews with 10 employees of the Swedish social insurance agency (Försäkringskassan, FK). We did secondary analysis of taped and transcribed formal interviews with 20 participants in a Swedish rehabilitation study [8,9] and 12 American employees of a public transportation company [10]. We examined data from expert group meetings, conferences, and literature data as well as quantitative data on sick leave in a cohort of 196 people. Participation by the first author in international grounded theory workshops 2003–2006 was a source of both interview data and memos. Table 1 shows characteristics of the participants.

**Table 1:** Characteristics of participants.

	Number of people	Age span (mean age)	Women/Men, %	Non-Swedes and immigrants	Education >12 years	Sick-listed/disability pension
Formal interviews	52	20–65	55/45	40%	40%*	60%
Informal interviews	72	20–80*	60/40*	25%*	80%*	20%*
Group interviews	10	30–60	80/20	0%	100%	0%
Quantitative sick leave data	196	21–62 (47)	50/50	19%	40%*	75%

*approximations

### Data analysis

We did classic grounded theory (GT) analysis according to Glaser [11-15] aiming at generating conceptual theories abstract of time, place and people. Classic GT differs from studies using qualitative data that often claim to be GT [16] by presenting explanatory concepts rather than descriptions. All of the data mentioned above was compared in the analysis according to the classic GT "all is data" dictum [12]. Field notes from interviews not taped were coded and compared in the same way as transcripts from taped interviews. All initial interviews were done with the "staying open" attitude of GT and the researcher being a "big ear" to the participant. We used no interview guides and posed no structured questions in the beginning of the research. In later stages of the study selective interviewing and coding was done with the emergent theory guiding our data collection. We also analysed the literature based on classic GT principles. A literature review was thus not done until central concepts of the theory had emerged through a cyclic process of collecting, coding, and comparing incidents in the data. Theoretical memos, in the shapes of text, diagrams, and figures, were written, typed, or drawn and several hundreds of pages of typed and handwritten memos sit in the memo bank from which this paper was sorted and written up. "Memos are the theorizing write-up of ideas about substantive codes and their theoretically coded relationships as they emerge during coding, collecting and analyzing data" [12]. Memos were eventually sorted and written up according to classic GT. We now compared relationships between categories and concepts using theoretical codes [11,13,15]. The writing of two working papers enhanced memo sorting: in 2005 for a Swedish research report [17] and in 2006 for a GT seminar in London, UK. The intensity of the analytic process increased over time. The theory was modified until the last writings of this article as a response to helpful peer reviewer suggestions. In summary, GT research is done in sequence, simultaneously, subsequently, serendipitously, but at the same time scheduled [12].

Many research methods consider persons or patients as units of analysis, whereas in GT the unit of analysis is the incident [18] of which the number often amounts to several hundred in a GT study since every participant often reports many incidents. When comparing many incidents in an area the emerging concepts and their relationships are in reality probability statements and thus classic GT is not a qualitative but a general method that can use any type of data [14]. The inductive nature of classic GT with hypotheses being generated has its roots in quantitative inductive research [12,19]. GTs are not reports of facts but integrated conceptual hypotheses based on empirical data. Validity is hence not an issue in GT research, which instead should be judged by fit, relevance, workability, and modifiability [12]. Fit has to do with how close concepts fit with the incidents they are representing. The theory works when it explains how the problem is solved with variation. A relevant theory fits, works, and deals with the real concern of participants and grabs attention. A modifiable theory can be altered when new relevant data is compared to existing data. A GT is never right or wrong, it just has more or less fit, relevance, workability and modifiability, and readers of this paper are asked to try its quality according to these principles.

The regional ethics committee at Lund University approved this study (LU-390-03), and interviews were made with informed consent from participants.

## Results

Reincentivizing is a theory explaining difficulties and strategies to help people go back to work after sick leave or other reasons for work absence.

### Understanding incentives, capacities, modes, drivers and traps

In this study drivers are specified as a combination of incentives and capacities to pursue a certain mode of action such as work. Traps are situations where drivers are locked in a certain mode. The notions of traps and drivers can explain many issues regarding disability and sick listing.

*"A driver is what makes you go on in a mode and a trap is what prevents you from getting out of it" *from theoretical memo

We define a ***driver ***as a combination of incentives or motivators and capacities. *Work capacities *are education, health, training, physical and psychological abilities, and social skills etc. Non-monetary *work incentives *are fellowship, identity, meaning, desire, plight, pride, and "flow" [20] but also shame-avoidance [21] etc. Monetary work incentives or non-work incentives are wage and sick leave compensation, unemployment benefits, fringe benefits or expenses such as meal costs, clothes, traveling, and time to do repairs of homes, cars etc.

A change in incentives or capacities can hurt mode drivers. Illness may hurt a work mode driver if the work mode capacity goes down. Eventually a hurt work driver may cause sick leave.

Hurt capacities and incentives eventually ***traps ***a person in a certain mode, (see below). In addition, time dependant inertia can trap mode drivers. This means that the longer a person has been in a certain mode the more difficult it is to change that mode, and thus the person gets trapped. Thus, if a person has been on sick leave for a long time it is difficult to go back to work since the inertia that comes from being in the sick leave mode for a certain time prevents the person from going back to work.

*"after three months of sick leave it is difficult for people to return to work" *physician, male middle aged

### Recognizing hurt work drivers and traps

Many ***driver assessments ***are done in sick leave situations. This either results in reincentivizing or disincentivizing a work return.

#### Mode driver calculation

Primarily, every person aims for her optimal "being mode" by an automatic mode driver calculation (Mdc), modified after Ekström (2005) [22]. A Mdc has three main outcomes: *preserving *a mode, *limiting losses *within a mode, and eventually *reevaluating *a mode. The Mdc weighs up mode incentives and capacitites in a cost-benefit calculus. Let's say an ill person is uncertain about being able to work since he/she feels depressed or suffers pain while working. So the work driver is hurt. Then mode *preserving *is first done: Enduring anxiety and pain by sticking to fundamental beliefs, strategies, and explanatory models. Keeping up habits, goals and daily life and continue working. Or the person goes on to *limiting losses*: Trying to stay in the mode as long as possible. Trying to master the situation by seeking knowledge, investing in life style changes, new health care contacts, or cutting down work, changing work tasks, taking short sick leaves or holidays. Or the person eventually *re-evaluates *the situation: Changing the mode by going on long sick leave, or changing job. If illness is severe enough the preserving and limiting stages are bypassed into immediate reevaluation.

Sick leave in itself can be seen as a mode with its own drivers. The Mdc basically determines whether an ill person works or stays at home. Ill health is then only one factor in the calculus. An ill person with an otherwise high work capacity combined with strong work incentives (monetary and non-monetary) has a strong work driver and a low risk of sick leave. Another ill person with an otherwise low work capacity and weak work incentives has a high risk of sick leave, See Table 2.

**Table 2:** Strength of work driver in relation to risk of sick leave

	High work capacity	Low work capacity
Strong work incentives	Strong work driver gives low risk of sick leave	Average work driver gives intermediate risk of sick leave
Weak work incentives	Average work driver gives intermediate risk of sick leave	Weak work driver gives high risk of sick leave

A 2×2 table presenting risk of sick leave as a function of degree of work capacity and work incentives.

Being modes affect incentives. While working the ill person primarily wants to preserve the work mode, but may eventually reevaluate the situation and go on sick leave. Having been on sick leave for some time the sick leave mode gets stronger through inertia. The Mdc now preserves another status quo and thus either reincentivizes work or chooses sick leave (see trapped mode drivers).

*Should I go on working despite my symptoms or stay at home? Should I return to work now as my symptoms are reduced or should I stay at home until they are completely gone? If I stay home from work what is the cost in terms of money and/or humiliation from my employer/fellow workers and/or the social insurance and what are the gains in terms of reduced suffering? *from theoretical memo

Other participants in the sick leave situation also make assessments and mode driver calculations:

#### Employer assessment

Employers may use a calculus similar to the Mdc when an employee turns ill. *Preserving *the existing situation is first done. This is followed by *limiting losses*, ie having the person cut down on tasks. Finally, the employer is *reevaluating *the situation, often by replacing the person by another employee, eventually permanentely. This replacement reevaluating strategy disincentivizes work return. But, if the employer regularly contacts absent employees and cooperates with the FK (social insurance) this can reincentivize work return. In rehabilitation planning the employer input is crucial for a work return.

#### Physician assessment

Physicians also calculate hurt work drivers in a physician assessment, which can either reincentivize or disincentivize work return. When writing sick leave notes physicians are either reincentivizing work return by being restrictive about sick leave:

*"You don't really need sick leave for this condition, you can actually go on working!"*middle aged male physician

or disincentivizing it by doing what the patient wants [23].

*"How long sick leave do you want *[*me to write in the sick leave note*]" (middle aged male physician).

#### Social insurance (FK) officials'assessments

When assessing requests for sick leave FK either reincentivizes work return by handling cases restrictively – *"Tiredness is not a reason for sick leave" *FK executive

or disincentivizes work return by speeding up "client" turn-over and promptly providing sick leave benefits:

*"The trick is to feed the PUMA (permanent and automatic benefit payment without control of sick leave status, abbreviated PUMA in Swedish)" *FK official.

So whether an ill person goes on sick leave depends on the Mdc, and assessments of employer, physicians and FK officials. But there is also a higher societal or macro level that determines sick leave behavior:

#### Macro level assessment

On the macro or society level the social insurance has three ways to go in the sick leave situation. Either *preserving *the present sick leave policies regarding legislation and compensation levels; or *limiting *sick leave by moderately restricting policies or by influencing attitudes towards sick leave; or *reappraising *the situation by radical changes of policies.

Another way of explaining sick leave and work absence is the notion of ***mode traps ***that are obstacles for reincentivizing. A person can get trapped in a certain mode of behavior through different (dis)incentivizors such as inertia, changing incentives or capacities. There are different drivers for different modes and these can trap the individual from reincentivizing work, ie from going back to work or go on working. Below are examples of traps associated with work and sickness absence and the reader may probably come to think of more trap types already.

#### Body trap

A person suffering pain or ill-health can be said to have a body-trapped work driver. This is the traditional reason for sick leave. The work incentives may be there, but they are locked in the hurt body. Basically, "body trap" means that your body prevents you from working. Work incentives could be high but body capacity is low.

*"It is like your body energy is trapped, you can barely handle everyday tasks and work is unthinkable" *middle-aged woman.

*"When body says no, work incentives are low" *middle-aged man

#### Sick role trap

This is common trap indicating that illness and sick leave can induce identity changes in a person so that the sickness mode becomes normal.

*"after two to three months of sickness absence the patient often gets stuck in a sickness role that is very difficult to get out of" *FK (social insurance) physician expert. Medicalization, ie when non-medical problems become medical issues also strengthens the sick role.

#### Poverty trap

Monetary disincentivizors in the Swedish labor market has been recognized by the government report "Out of the poverty trap" [24]. In Sweden it is difficult for persons on long-term sick leave with a low income to increase their income by returning to work due to marginal effects of the social security system. These marginal effects are disincentivizing work by reincentivizing non-work modes. Also, the poverty trap often brings on dehumanizing side effects of being outside of society and the work force such as impaired health and lowered self confidence.

#### Fox trap

A person on sick leave having a limited work capacity belongs neither in the work mode nor in the sick leave mode. Instead of being on full time sick leave or working full time despite illness a person can be on partial time sick leave (common in Sweden). However, having no employement the person is in the fox trap – *"you are too healthy to be on sick leave" *says the FK official, while the employment service agent says *"you are too ill to be working".*

#### Honey trap

This mode trap may cause future work absence. Too much stimulation by strong work incentives, both monetary and non-monetary will eventually trap a person in a high pace work mode difficult to get out of. Eventually this causes illness and a limited future work capacity. People working with creative tasks thus risk getting stuck in the honey trap. Family life and leisure are annoying breaks in work, which becomes the primary meaning of life. The honey trap involves a reincentivizing positive feedback mechanism. The incentive makes you work more, which gives more incentive, and finally you cannot stop working at a pace that is too high for your capacities. Eventually the work driver is hurt.

"*At X the honey trap is a fact. People get here from all over the world. They love their work – solving problems etc, and if they don't watch out they get stuck in the (honey) trap..." *Middle aged employee with creative job in multinational company.

#### System trappers

Some people in welfare states take advantage of the compensations in the welfare system. One might say that they are "working the system", and we may call them system "trappers" since their behavior could be compared to that of hunters and gatherers. System trappers are found in every society with a welfare system but seem especially common in areas where people traditionally make their living from hunting, fishing, and forestry, and where regular jobs are limited. In scarsely populated parts of Canada it is considered socially acceptable to do paid work as little as possible and rely on social security as an important monetary support. Similarly, in Sweden scarsely populated areas have the highest number of people on sick leave and unemployement benefits. Attitudes towards such benefits in these areas are less linked to shame than in other areas with a stronger labor market. Also in Bolivia, the native population collect (in comparison to western standards) limited welfare subsidies in the same way as they collect natural resources through hunting, fishing and gathering. So, even if work incentives seem to vary culturally, geographically and demographically, there are important similarities.

### Reincentivizing work by driver repair and trap release

Reincentivizing is done by repairing hurt work drivers, ie hurt capacities and incentives for work. When drivers are hurt they need repair, and by repairing them the traps get released. The different repair strategies are linked to each other in a multitude of ways and often occur simultaneously.

### Driver repair – capacity, non-monetary and monetary incentive repair

Reincentivizing by improving the health and well being of a person on sick leave is fundamental. We call a first and almost self-evidentiary aspect of it body repair.

#### Body repairs

for impaired body capacities are medication, physiotherapy, surgery, rehabilitation programs [8,9] and alternative therapies. Succesful treatments eventually reincentivize the work return. Irrepairable illness often leads to disability compensation such as a disability pension.

#### Self repair

Socializing with friends and relatives, having pets, physical exercise, and hobbies may enhance non-monetary incentives and restore work capacity at the same time. This is achieved by an improved well-being which ameliorates work return. However, long duration of self-repair activities might weaken the work driver since long time away from work disincentivizes work return through the inertia of being in a non-work mode.

#### Work-place repair

Making the work place a better environment for the employee can reincentivize work. Bad management may cause emotional strains that risks eroding work identity, a powerful work incentive. It is therefore important that supervisors try to create a positive emotional atmosphere [25]. Structured back-to-work programs where absent employees are contacted and fellow workers informed of possible changes in task assignments when the absentee returns are also beneficial. It seems as the more employers are engaged in rehabilitation programs the more work can be reincentivized [25].

#### Rehumanizing

Strengthening non-monetary work incentives and thus increasing work capacity can prevent a person from going on sick leave. This can be achieved by joining support networks in the workplace that may startle a rehumanizing process [26] promoting authenticity, safety and healing. By giving network members challenge, experimentation, and creativity, this can provide the worker with new energy and learning.

#### Controlling sick leave insurance

There are three main ways to reincentivize work by controlling the sick leave insurance. First, making it more difficult to obtain by controlling its eligibility. Second, controling non-monetary incentives, and third making it less financially beneficial to be on sick leave.

##### Controlling insurance eligibility

Reincentivizing would be enhanced by a stricter control of the sick leave insurance eligibility, which has been characterized as being too "soft". A stricter control means that FK and employer assessments have to be tougher. Hence, the trust in the Mdc and physician assessment is often reduced. A 2006 government report suggests the use of Medical Disability Advisor (MDA) guidelines from the USA for limiting the length of sick leave periods [27].

##### Controlling non-monetary incentives

Shame, fear and plight could disincentivize sick leave. In national multimedia ad campaigns FK linked sick leave to shameful behavior and subtle fraud. Hence, by inflicting shame, and appealing to societal plight people would become less prone to go on sick leave.

*"it (the ad campaign) puts a sick leave controller in the head of the person on sick leave" *regional FK CEO.

In a postal intervention study to newly sick-listed persons sick leave periods of >12 weeks were reduced for the group receiving brief information and a short questionnaire [28].

##### Controlling monetary compensation

Hurt monetary incentives disincentivizes a return to work for those who have been on sick leave long enough to trust the monthly payments from the FK. By cutting down monetary compensation levels of sick leave (and of unemployment benefits) it is possible to reincentivize work [3,29].

*"Sick leave would probably go down if compensation levels were lowered..." *former national FK CEO

#### Strengthening monetary work incentives

Making work monetarily advantageous in relation to non-work could be done on the macro level by using tax policies. In the UK working families get a special tax deduction as compared to families on welfare. In Sweden the new 2006 government launched a tax deduction eligible only for workers, not for people on sick leave or retirement pension.

### Trap release

Traps are essentially released by the above repair strategies. Either body and/or work place repair can release from a *body trap*. Improving impaired health situations and work place conditions can help workers with health problems to return to work. Controlling sick leave insurance and strengthening *monetary work incentives *might get people out of the *poverty trap*. By all three strategies a *sick role trap *or a *fox trap *can be released. Education or job training programs could release from the *fox trap *by increasing work capacity. A *Honey trap *can be prevented by self repair or work place repair. Some employers are aware of honey-traps and prevent their employees from getting consumed by overmotivating jobs. They sense signals of overstimulation and require that employees take time off. So a release from the honey-trap can be done through an initiative from the employer or another person in order to prevent a future damage to the work driver. *System trappers *can be controled by sick leave insurance repair. A stricter control of eligibility and reduced monetary compensation of different types of social insurance will prevent people from abusing the welfare system.

## Discussion

In this study of work absence and sick leave we present a theory explaining why it may be difficult to return to work after sick leave, and what can be done to ease the return. Reincentivizing indicates that work incentives, both monetary and non-monetary, and not only health related factors are important in the process of a work return [30]. Reincentivizing is a theoretical model that fits with the wide range of data from which it was generated. It also works to explain many work and sick leave related issues. The model applies to the Swedish situation with one of the highest sickness absence rates in the world, but we believe that reincentivizing is relevant for other settings as well. To understand reincentivizing we start with specifying concepts that are central to the theory. Then follows recognizing hurt work drivers and traps. Third, reincentivizing is done by repairing hurt capacities and incentives, and releasing from traps.

We did classic grounded theory (GT) analysis according to Glaser [11-15] to develop the reincentivizing theoretical model. We interviewed people working or on sick leave as well as physicians and social insurance officials, and also analyzed literature. Our procedure was similar to previous studies in other substantive areas [31,32]. GT is the most cited single method for analyzing qualitative data according to Google Scholar where the first GT methodology [18] had 6995 citations in May 2007. Yet classic GT studies are rare. They represented <10% of 200 consecutive studies referring to the method in a PubMed search in 2005–2006 done by the first author. Most studies were descriptive and lacked a core variable theory, which is required in classic GT.

The concept driver is fundamental to reincentivizing and commonly used in contemporary Swedish language: "what is your driver?" "what is the driver in your life...". In GT this is called an in-vivo code, ie it comes from the participants in the interview data. Trap is another in-vivo code from the area of sick leave used by unions, employer organizations, and government agencies. The body trap is another way of expressing work absence due to illness – the worker is trapped in a harmed body. To be in a honey trap resembles the colloquial expression "workaholic". Poverty trap is a concept borrowed from a Swedish government report [24] and sociology. A similar concept is called "low pay traps" that are disincentives for people to stay in the workforce [33]. Fox trap is a concept found in a white-collar workers union report. The mode driver calculation (Mdc) is a concept generated by inspiration from two grounded theories – "Cutting back after a heart attack" [34], "Keeping my ways of being" [22] and Jeremy Bentham's "hedonic calculus" [35]. Mullen suggested that people having suffered a heart attack "cut back" in their lives after a complex calculus. Ekström proposed that women in midlife apply a personal calculus to keep up their way of being when faced with insecurity caused by midlife changes. Jeremy Bentham in 1798 claimed that every person was aiming for ultimate happiness by applying a "hedonic calculus" in life: "promoting whatever factors led to the increase of pleasure and suppressing those which produced pain".

Our theory of reincentivizing work fits in the literature on work, sickness absence and unemployment in several diverse fields such as sociology, economics and medicine [1-4,7-9,21,29,30,35,36]. It attempts to integrate previous research findings together with new empirical data in an explanation of what motivates complex fundamental human behavior such as work. Theoretical explanatory models for sick leave behavior are scarce. A process model explaining absenteeism with data from the USA has been presented [37]. It includes different variables such as work-related attitudes, personal factors, market factors and cultural and organizational norms in an organizing framework for understanding absence research. Our theory of reincentivizing seems to fit into that framework, yet with a more parsimonious explanation. Historically work incentives seem to be about balancing between working for a greater good such as society or God, and working for profit (apart from working for supporting basic life processes). In our study the non-monetary and monetary incentives for working represent this balance. In typical welfare states such as Sweden plight incentives are stronger than in laissez-faire economies such as USA [2]. This is reflected in high Swedish compensation levels and a weak control system for sick leave. The Swedish expression "writing your own sick leave note" typically indicates the ease by which sick leave may be attained in this country. Societal stability motives for having generous sick leave policies – possible reduced costs of health care, basic social welfare, policing, and drug control – could legitimize the present high compensation levels. But between 1997 and 2003 both unemployment and sickness absence increased in Sweden to levels allegedly threatening the working morale of the population and eventually the foundation of the welfare state [3]. Hence, a crucial issue in the 2006 parliament election campaign was to reduce the high number of people outside of the work force. This led to the first shift in government for 12 years with the new government suggesting lowered compensation for sick leave and unemployment. This was a political risk taking since 14% of the Swedish population depends on sick leave insurance or disability pension for their daily living [3]. A November 2006 government report suggested stricter sick leave assessments using the length of mean sickness absence periods in the USA as a standard [27].

It may be argued that the value of our study is limited since it is not traditionally deductive. Neither is it a full description of sick leave or work absence phenomena. It is rather inductive since GT is primarily an inductive method. Reincentivizing work is according to GT a theory with a certain degree of probability and ability to provide an explanatory account of the area under study. It is not a presentation of proven facts but a suggested conceptual explanation of what is going on in the area of work and sick leave. We admit that we have missed data in our comparative analysis of sick leave and work. We did for instance not study self-employed people. Yet there is Swedish data showing lower odds of sick leave in self-employed despite more subjective illness as compared to matched controls [38]. However, we trust that our theory is modifiable when "missing" data is entered into the analysis. Thus, by adding new data more concepts will eventually be generated that will add to the theory, not contradict it. We therefore encourage readers to pursue research in this field, and refine and improve the suggested theory of reincentivizing work.

## Conclusion

We have developed a theory suggesting that complex drivers determine people's behavior. These drivers can work either to incentivize or to reincentivize different modes. To deal with sick leave according to the theory of reincentivizing work first requires an understanding of the concepts of drivers and traps. Then hurt drivers and traps are recognized and eventually repaired and released. The theory of reincentivizing work could give ideas for future research, and possibly inform changes in sick leave policies.

## Competing interests

The author(s) declare that they have no competing interests.

## Authors' contributions

HT and BG together conceived, designed, and collected data for the study. HT did the grounded theory analysis and drafted the manuscript in collaboration with BG. Both authors read and approved the final manuscript.

## Pre-publication history

The pre-publication history for this paper can be accessed here:


